# Supplementary material for: Loss of α2-6 sialylation promotes the transformation of synovial fibroblasts into a pro-inflammatory phenotype in arthritis
Source: Nat Commun. 2021 Apr 20;12:2343. doi: 10.1038/s41467-021-22365-z (PMC8058094; doi:10.1038/s41467-021-22365-z)
Supplement: Supplementary file 1 — Supplementary Information [file 41467_2021_22365_MOESM1_ESM.pdf]

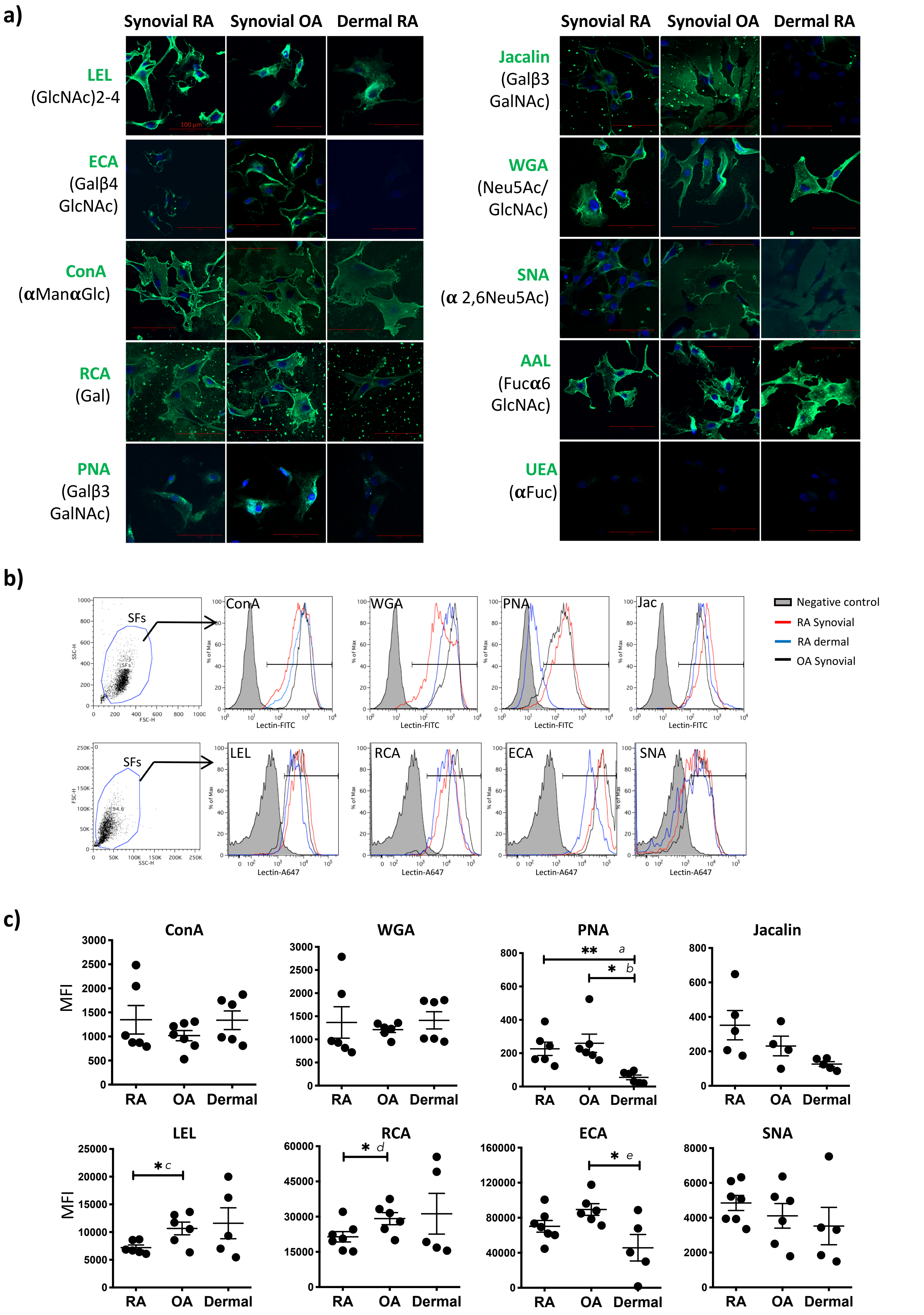

a)
Synovial RA
Synovial OA
Dermal RA
LEL (GlcNAc)2-4
ECA (Galβ4 GlcNAc)
ConA (αManαGlc)
RCA (Gal)
PNA (Galβ3 GalNAc)
Jacalin (Galβ3 GalNAc)
WGA (Neu5Ac/ GlcNAc)
SNA (α 2,6Neu5Ac)
AAL (Fucα6 GlcNAc)
UEA (αFuc)
100 µm
b)
SFs
ConA
WGA
PNA
Jac
LEL
RCA
ECA
SNA
Lectin-FITC
Lectin-A647
SSC-H
FSC-H
% of Max
Negative control
RA Synovial
RA dermal
OA Synovial
c)
ConA
WGA
PNA
Jacalin
LEL
RCA
ECA
SNA
MFI
RA
OA
Dermal
** a
* b
* c
* d
* e

Supplementary Figure 1: Glycosylation of human fibroblasts varies in distinct anatomical locations and inflammatory environments. a) Immunofluorescence staining (lectin, green) and nuclei (DAPI, blue) of synovial and dermal fibroblasts isolated from RA patients following joint replacement surgery, and synovial fibroblasts isolated from OA patients. Lectins and their carbohydrate binding specify are shown. Scale bars = 100 µm. Experiment was repeated twice with similar results. b) Flow-cytometric analysis of indicated lectin staining on non-permeabilised synovial fibroblasts (RA) and dermal fibroblasts (Dermal) from representative RA patients, and synovial fibroblasts from osteoarthritis patients (OA). c) Mean Fluorescence Intensity (MFI) of the whole population showing individual patients. Statistics: Data are presented as mean ± SEM, n= [RA: (Jacalin, n= 5; ConA, WGA, PNA, and LEL n=6; RCA, ECA and SNA n=7. OA: Jacalin n=4; WGA, PNA, LEL, RCA, ECA and SNA n=6; WGA n=7). Dermal: (Jacalin, LEL, RCA, ECA and SNA n=5; ConA, WGA and PNA n=6.)] individual patients. Statistical significance was determined using Brown-Forsythe and Welch ANOVA test for multiple comparisons and significance indicated by asterisks, * $p < 0.05$ and ** $p < 0.01$. Actual p values: *a*: 0.0062, *b*: 0.0121, *c*: 0.0281, *d*: 0.0436, *e*: 0.0412.

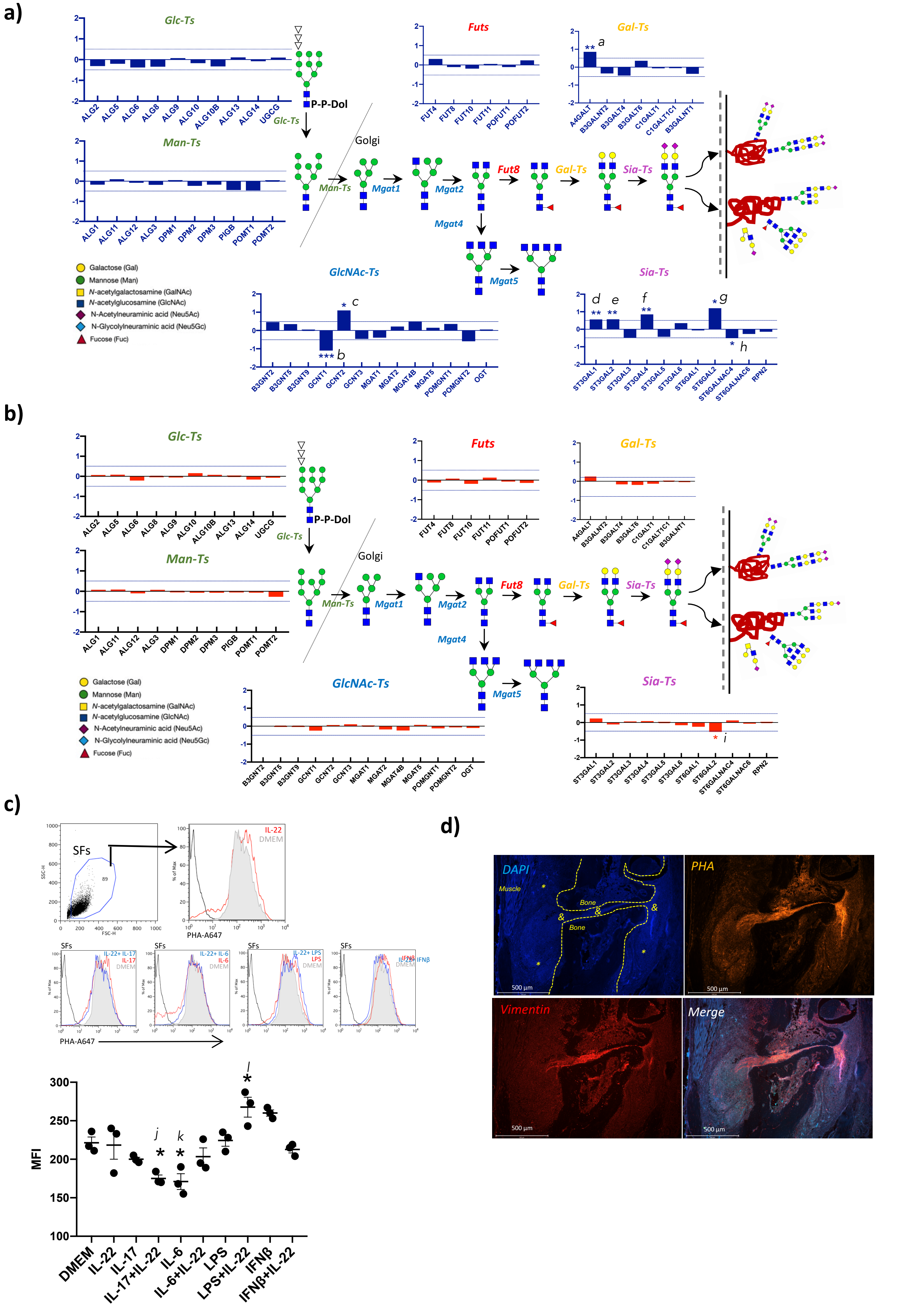

a)
Glc-Ts
ALG2
ALG5
ALG6
ALG8
ALG9
ALG10
ALG10B
ALG13
ALG14
UGCG
P-P-Dol
Man-Ts
ALG1
ALG11
ALG12
ALG3
DPM1
DPM2
DPM3
PIGB
POMT1
POMT2
Golgi
Mgat1
Mgat2
Mgat4
Mgat5
Fut8
Gal-Ts
Sia-Ts
Futs
FUT4
FUT8
FUT10
FUT11
POFUT1
POFUT2
A4GALT
B3GALNT2
B3GALT4
B3GALT6
C1GALT1
C1GALT1C1
B3GALNT1
GlcNAc-Ts
B3GNT2
B3GNT5
B3GNT9
GCNT1
GCNT2
GCNT3
MGAT1
MGAT2
MGAT4B
MGAT5
POMGNT1
POMGNT2
OGT
ST3GAL1
ST3GAL2
ST3GAL3
ST3GAL4
ST3GAL5
ST3GAL6
ST6GAL1
ST6GAL2
ST6GALNAC4
ST6GALNAC6
RPN2
Galactose (Gal)
Mannose (Man)
N-acetylgalactosamine (GalNAc)
N-acetylglucosamine (GlcNAc)
N-Acetylneuraminic acid (Neu5Ac)
N-Glycolylneuraminic acid (Neu5Gc)
Fucose (Fuc)
b)
c)
SFs
89
SSC-H
FSC-H
IL-22
DMEM
PHA-A647
% of Max
IL-22+ IL-17
IL-17
IL-22+ IL-6
IL-6
IL-22+ LPS
LPS
IFNβ
IL-22+ IFNβ
MFI
DMEM
IL-22
IL-17
IL-17+IL-22
IL-6
IL-6+IL-22
LPS
LPS+IL-22
IFNβ
IFNβ+IL-22
d)
DAPI
Muscle
Bone
PHA
Vimentin
Merge
500 µm

Supplementary Figure 2: Cytokines regulate glycosylation in synovial fibroblasts. a-b) Gene expression of human SFs following stimulation with TNF, 1 ng/ml, 6h (a) and IL-17, 10 ng/ml, 6h (b). Original data-set was generated by Slowikowski et al[25], to assay transcriptomes of seven synovial fibroblast cell lines (RA=4, OA=3). Expression of glycosyltransferase genes was analyzed in the publicly available resource https://immunogenomics.io/fibrotime, where authors used Kallisto and Limma R package to test for differential gene expression. Glycosyltransferases genes were selected and genes that pass a threshold of $p < 0.05$ in DE analysis are shown, *$p < 0.05$ and **$p < 0.01$. Actual p values: *a*: 0.0018, *b*: 0.00033, *c*: 0.045, *d*: 0.0066, *e*: 0.089, *f*: 0.0019, *g*: 0.012, *h*: 0.005, *i*: 0.0088. c) Murine SFs were isolated and expanded from mouse synovium and incubated with the indicated inflammatory mediators (10 ng/ml for IL-22, IL-17, IL-6, and IFNβ and 0.2 μg/ml for LPS) for 24 hours, when binding of PHA was evaluated by Flow Cytometry. Statistical significance was determined using ordinary one-way ANOVA with Bonferroni's multiple comparisons test, *$p < 0.05$. Actual p values: *j*: 0.0241, *k*: 0.0121, *l*: 0.0241. d) Joints of CIA mice show an association of PHA binding (multi-branched glycans) and stromal cells in inflamed areas. Bone is shown with dotted yellow lines, (*) immune cell infiltration, (&) stromal-mediated inflammation, (#) synovial fibroblast expansion. Stromal marker vimentin (red), PHA binding (orange) and DAPI staining (blue) are shown. Scale bars: 500 μm. Experiment was repeated independently in three animals with similar results.

a)

Top 50 up-regulated genes					
**Gene ID**	**Fold increase**	**padj**	**Gene ID**	**Fold increase**	**padj**
Il1rn	555.47	3.10E-11	Mmp3	11.08	5.62E-25
Olfm4	540.04	2.33E-10	Fcgr2b	11.04	6.34E-05
Il18rap	387.82	1.55E-08	Acod1	10.57	2.18E-07
Reg3g	335.02	5.42E-11	Lcp1	10.50	4.96E-08
Saa3	129.99	3.48E-32	Timp1	9.05	1.20E-99
Cxcl5	128.37	8.51E-96	Col10a1	8.91	1.71E-06
Csf3	125.00	2.25E-04	Ptprn	8.78	1.46E-07
C430002N11Rik	117.00	1.66E-06	Lcn2	8.68	3.68E-84
Cxcl3	99.26	2.77E-03	Saa2	8.37	7.47E-04
Prokr2	77.84	7.79E-16	Bank1	8.32	6.30E-04
Csf3r	58.00	4.23E-07	Rtn4rl2	8.28	1.78E-07
5730559C18Rik	37.45	2.43E-13	Gja4	8.03	6.76E-06
Uox	32.11	1.08E-09	Apln	7.99	3.48E-32
Il1b	24.24	1.44E-10	Kif26b	7.97	8.36E-14
Ccl20	22.85	6.62E-03	Cdca7l	7.65	1.23E-06
Gm44439	21.37	3.08E-04	Chl1	7.55	3.46E-10
Lilr4b	18.45	7.76E-05	Gm48878	7.53	1.24E-07
Acat3	16.42	7.28E-26	Fam110c	7.27	4.90E-03
Hp	15.43	9.06E-23	Hcar2	6.98	4.09E-07
Spink6	13.09	1.21E-09	Ptx3	6.95	5.28E-59
Lman1l	13.08	1.38E-13	Cenpm	6.84	3.91E-03
Hdc	12.85	5.96E-06	Gpr39	6.36	4.86E-05
Ephx3	12.20	3.08E-07	Ccnb2	6.13	4.98E-12
Nlrp3	11.43	5.81E-03	Slc15a3	6.13	1.50E-08
Saa1	11.10	5.65E-32	Mogat2	6.09	1.27E-05

Top 50 down-regulated genes					
**Gene ID**	**Fold increase**	**padj**	**Gene ID**	**Fold increase**	**padj**
Tll2	0.18	3.46E-06	Sv2c	0.37	2.87E-06
Chp2	0.19	3.44E-05	430402I18Ri	0.37	7.03E-03
Podxl	0.20	2.34E-04	Hrh2	0.37	3.96E-06
A2m	0.21	1.41E-05	Abca6	0.38	8.38E-10
Rtl9	0.22	8.13E-04	Cdh19	0.38	8.80E-03
Cd300lg	0.24	4.66E-04	Tdrp	0.39	4.68E-03
Prrt4	0.24	1.73E-09	Fmo2	0.41	1.09E-11
Vsig8	0.25	3.53E-04	Gpr165	0.41	1.52E-04
F730311O21Rik	0.25	1.91E-03	Bmp3	0.41	3.89E-06
Ptpro	0.26	3.46E-03	Glt8d2	0.42	3.36E-18
Rtn1	0.26	3.76E-06	Mfap3l	0.42	1.56E-12
Exph5	0.27	1.98E-03	Tek	0.42	9.59E-08
Reln	0.29	1.10E-12	C7	0.43	3.25E-03
Gsta3	0.29	1.40E-07	Opcml	0.43	1.72E-05
Pcdh20	0.30	1.35E-03	Tenm2	0.43	4.89E-03
Stmn4	0.31	3.32E-06	Dact2	0.43	3.85E-05
Fzd10	0.32	8.96E-03	700019D03Ri	0.43	3.67E-07
Gabra4	0.35	6.19E-04	Gm11816	0.43	1.26E-03
Necab1	0.35	3.35E-03	Tppp	0.43	2.67E-03
Prom1	0.36	7.43E-04	Dpp4	0.43	6.08E-03
Ces1d	0.36	4.63E-03	Nexmif	0.44	8.03E-03
Lyve1	0.36	3.25E-03	Gpld1	0.44	8.55E-06
Col4a3	0.36	1.22E-05	Npr3	0.44	3.12E-06
Syt7	0.37	1.77E-05	Irf4	0.44	2.54E-03
Npy4r	0.37	2.98E-03	Tmod2	0.44	3.00E-10

b)

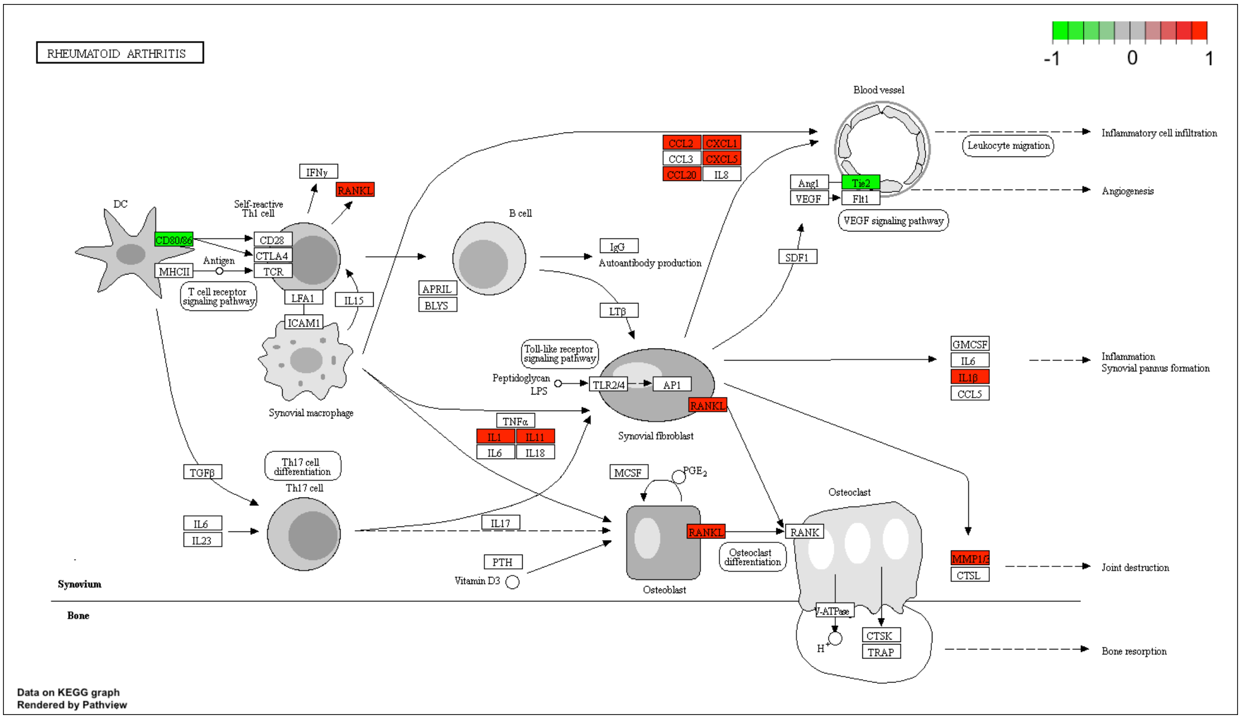

Supplementary Figure 3: Analysis of RNA-Seq data in Rheumatoid Arthritis disease pathway. a) Top 50 DE genes (padj < 0.01, as calculated for figure 2) in CIA SFs compared to healthy controls are shown in red (up-regulated) or green (down-regulated). b) RA disease pathway as defined by the KEGG database. Genes with significantly differential expression are shown for each gene, upregulated (red) and down-regulated (green).

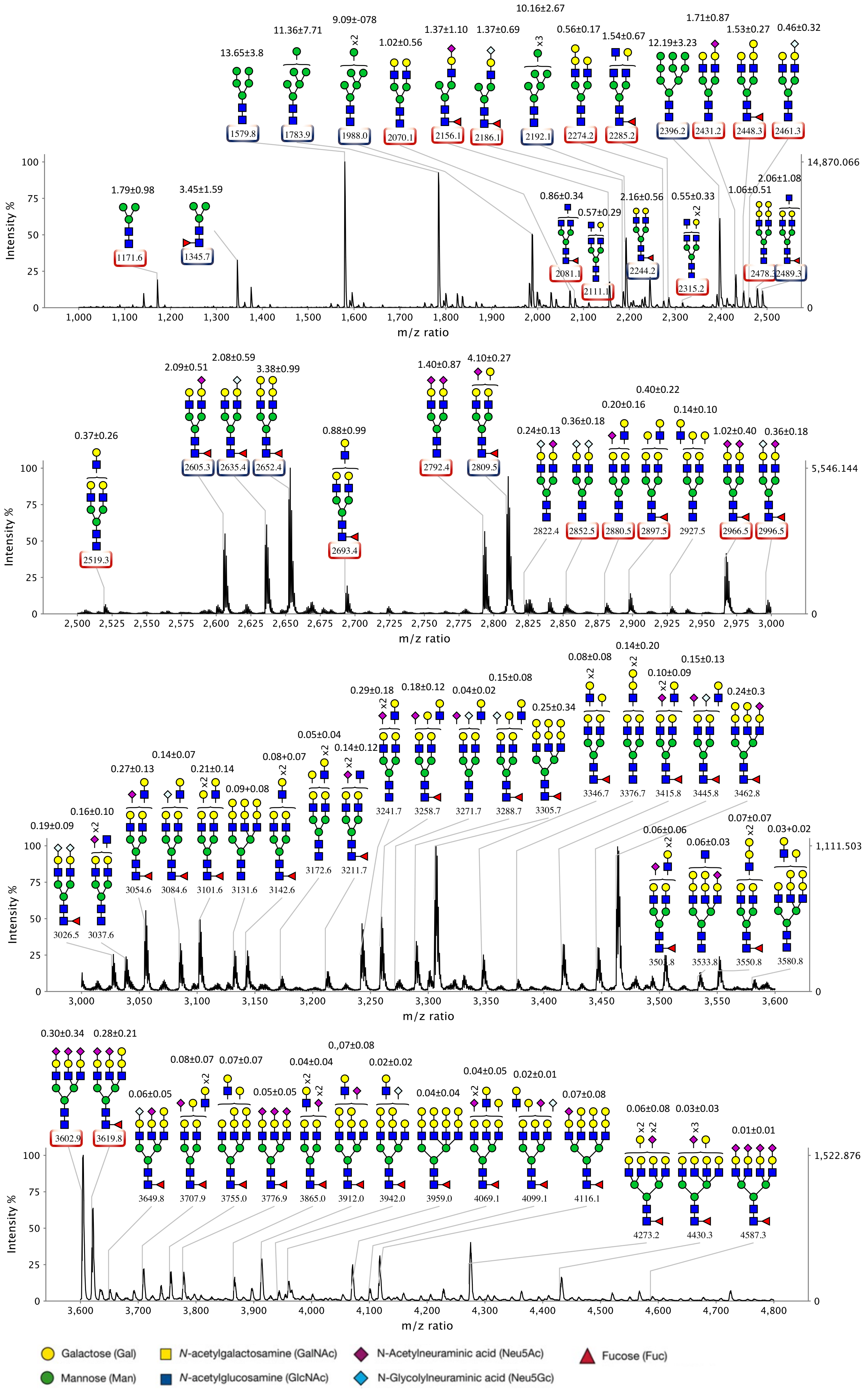
13.65±3.8
11.36±7.71
9.09±-078
1.02±0.56
1.37±1.10
1.37±0.69
10.16±2.67
0.56±0.17
1.54±0.67
12.19±3.23
1.71±0.87
1.53±0.27
0.46±0.32
1579.8
1783.9
1988.0
2070.1
2156.1
2186.1
2192.1
2274.2
2285.2
2396.2
2431.2
2448.3
2461.3
14,870.066
1.79±0.98
3.45±1.59
1171.6
1345.7
0.86±0.34
0.57±0.29
2.16±0.56
0.55±0.33
1.06±0.51
2.06±1.08
2081.1
2111.1
2244.2
2315.2
2478.3
2489.3
Intensity %
m/z ratio
2.09±0.51
2.08±0.59
3.38±0.99
1.40±0.87
4.10±0.27
0.37±0.26
0.88±0.99
0.24±0.13
0.36±0.18
0.20±0.16
0.40±0.22
0.14±0.10
1.02±0.40
0.36±0.18
2605.3
2635.4
2652.4
2792.4
2809.5
2519.3
2693.4
2822.4
2852.5
2880.5
2897.5
2927.5
2966.5
2996.5
5,546.144
0.29±0.18
0.18±0.12
0.04±0.02
0.15±0.08
0.25±0.34
0.08±0.08
0.14±0.20
0.10±0.09
0.15±0.13
0.24±0.3
0.19±0.09
0.16±0.10
0.27±0.13
0.14±0.07
0.21±0.14
0.09±0.08
0.08+0.07
0.05±0.04
0.14±0.12
3241.7
3258.7
3271.7
3288.7
3305.7
3346.7
3376.7
3415.8
3445.8
3462.8
3026.5
3037.6
3054.6
3084.6
3101.6
3131.6
3142.6
3172.6
3211.7
0.06±0.06
0.06±0.03
0.07±0.07
0.03+0.02
3501.8
3533.8
3550.8
3580.8
1,111.503
0.30±0.34
0.28±0.21
3602.9
3619.8
0.06±0.05
0.08±0.07
0.07±0.07
0.05±0.05
0.04±0.04
0.,07±0.08
0.02±0.02
0.04±0.04
0.04±0.05
0.02±0.01
0.07±0.08
0.06±0.08
0.03±0.03
0.01±0.01
3649.8
3707.9
3755.0
3776.9
3865.0
3912.0
3942.0
3959.0
4069.1
4099.1
4116.1
4273.2
4430.3
4587.3
1,522.876
Galactose (Gal)
Mannose (Man)
N-acetylgalactosamine (GalNAc)
N-acetylglucosamine (GlcNAc)
N-Acetylneuraminic acid (Neu5Ac)
N-Glycolylneuraminic acid (Neu5Gc)
Fucose (Fuc)

Supplementary Figure 4: MALDI-TOF MS profiles of the permethylated N-linked glycans derived from expanded murine SFs. Data were obtained from the 50% MeCN fraction, and all molecular ions are present in sodiated form ([M+ Na]+). Numbers above structures indicate the mean of relative expression ± SD (n=4 experiments). Numbers below structures show the m/z ratio. Blue rectangles show the structures constituting 75% of all the glycome and red rectangles show those completing 90% of total expression.

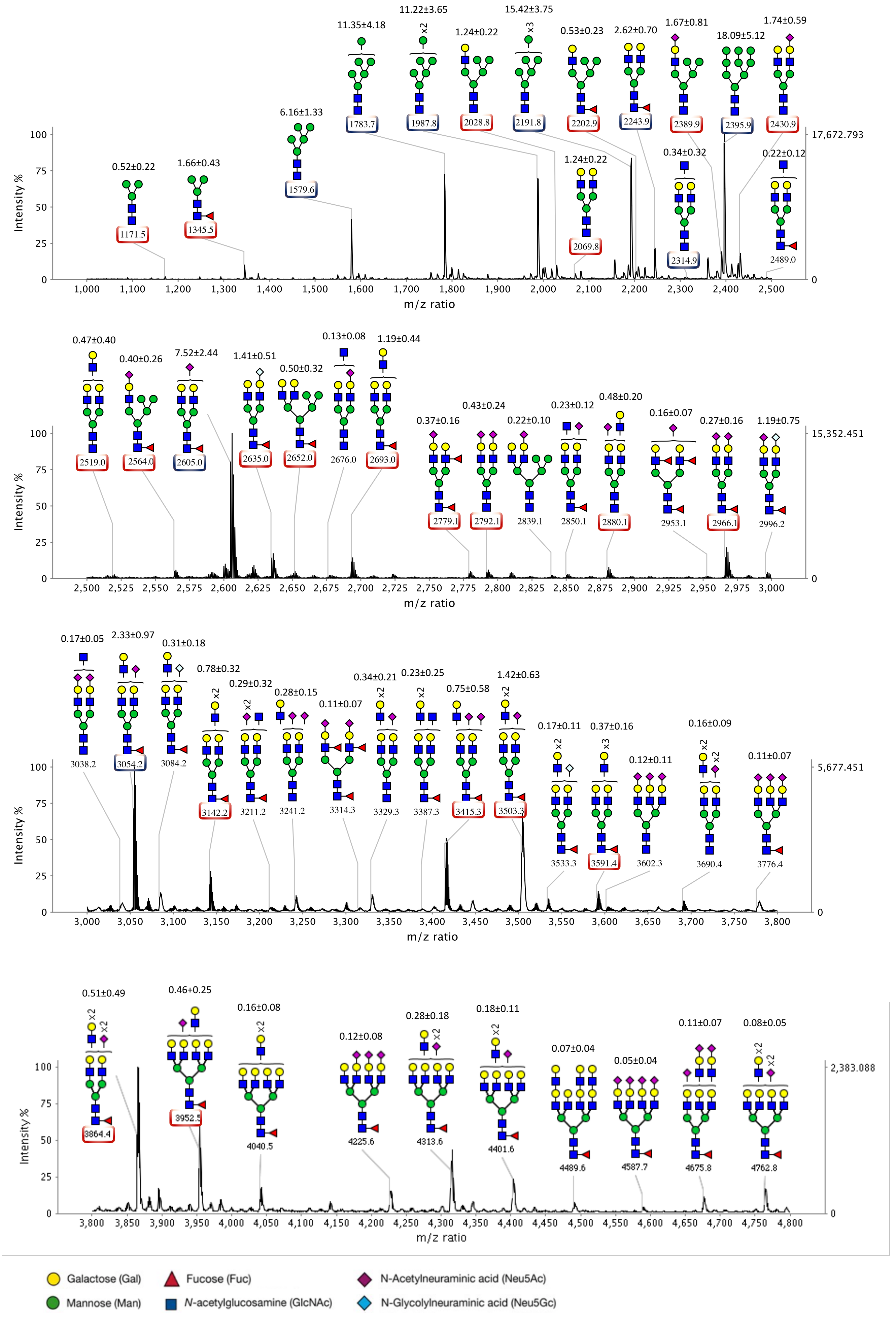

Intensity %
m/z ratio
17,672.793
15,352.451
5,677.451
2,383.088
1171.5
0.52±0.22
1345.5
1.66±0.43
1579.6
6.16±1.33
1783.7
11.35±4.18
1987.8
11.22±3.65
2028.8
1.24±0.22
2191.8
15.42±3.75
2202.9
0.53±0.23
2243.9
2.62±0.70
2389.9
1.67±0.81
2395.9
18.09±5.12
2430.9
1.74±0.59
2069.8
1.24±0.22
2314.9
0.34±0.32
2489.0
0.22±0.12
2519.0
0.47±0.40
2564.0
0.40±0.26
2605.0
7.52±2.44
2635.0
1.41±0.51
2652.0
0.50±0.32
2676.0
0.13±0.08
2693.0
1.19±0.44
2779.1
0.37±0.16
2792.1
0.43±0.24
2839.1
0.22±0.10
2850.1
0.23±0.12
2880.1
0.48±0.20
2953.1
0.16±0.07
2966.1
0.27±0.16
2996.2
1.19±0.75
3038.2
0.17±0.05
3054.2
2.33±0.97
3084.2
0.31±0.18
3142.2
0.78±0.32
3211.2
0.29±0.32
3241.2
0.28±0.15
3314.3
0.11±0.07
3329.3
0.34±0.21
3387.3
0.23±0.25
3415.3
0.75±0.58
3503.3
1.42±0.63
3533.3
0.17±0.11
3591.4
0.37±0.16
3602.3
0.12±0.11
3690.4
0.16±0.09
3776.4
0.11±0.07
3864.4
0.51±0.49
3952.5
0.46+0.25
4040.5
0.16±0.08
4225.6
0.12±0.08
4313.6
0.28±0.18
4401.6
0.18±0.11
4489.6
0.07±0.04
4587.7
0.05±0.04
4675.8
0.11±0.07
4762.8
0.08±0.05
Galactose (Gal)
Mannose (Man)
Fucose (Fuc)
N-acetylglucosamine (GlcNAc)
N-Acetylneuraminic acid (Neu5Ac)
N-Glycolylneuraminic acid (Neu5Gc)

Supplementary Figure 5: MALDI-TOF MS profiles of the permethylated N-linked glycans derived from expanded human SFs. Cells were expanded from synovial tissue isolated from OA patients. Data were obtained from the 50% MeCN fraction, and all molecular ions are present in sodiated form ([M + Na]+). Numbers above structures indicate the mean of relative expression ± SD (n=3 individual patients). Numbers below structures show the m/z ratio. Blue rectangles show the structures constituting 75% of all the glycome and red rectangles show those completing 90% of total expression.

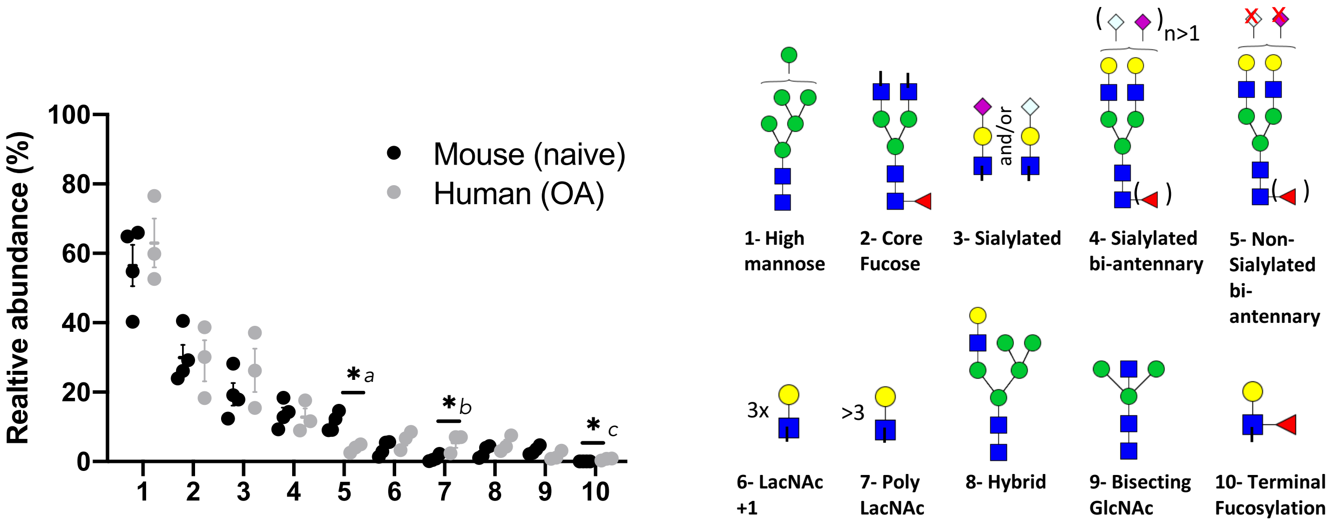

Supplementary Figure 6: Comparison of N-glycan structures between human OA and murine SFs. Structures shown in supplementary figure 4 and supplementary figure 5 were grouped into structurally related groups. Groups are shown and labelled from 1 to 10. Relative intensity for each group was summed and plotted. Each dot represents one individual experiment (murine cells, n=4; human OA cells, n=3), data are presented as mean ± SEM. Statistical difference was calculated using two-tail t test, $*p < 0.05$, $*p < 0.01$. Actual p values: *a*: 0.0072, *b*: 0.0219, *c*: 0.0076.

Name	Sequence : (5' to 3')
FM1_St3gal3	GGATCAAAGGTCAAGACAATC
RM1_St3gal3	CAATGTCATAGTCGTCAATCC
FM1_St3gal4	GATTGAGACCATCTTGAGTG
RM1_St3gal4	CTTCTGCTTGATCTTTCGAG
FM1_St6galnac2	CTACCTGACAGAGAGGTTTC
RM1_St6galnac2	GTCTGAGTATTTCTGGTAGTTG
FM1_St6galnac4	AGATCTTCCAAGATGAGACAG
RM1_St6galnac4	TTCTCAAAGTAGTGGTAGGG
FM1_St8sia3	CGTAACAACTTCTTCCTCAG
RM1_St8sia3	TGTTTTTCCAGTACCTGTTG
FM1_St3gal6	AGAGTATTTCCCAAGGATCAG
RM1_St3gal6	CATTCCCGTAGTAGTGTAAAG
FM1_St8sia6	GAGCGCTAGTAAAGATGTTG
RM1_St8sia6	CAAATTCTGGTACTTCAGGG
FM1_St6galnac6	GAAGACTCCCATGATGAAAC
RM1_St6galnac6	AAAGAGGATCACAAACACTG
FM1_Cmas	GACTGTAAAACAGAAGTCAGTG
RM1_Cmas	TTCATCAGACACTTCATTGC
FM1_Slc35a1	GTACCTACTTGTCAGATGGAG
RM1_Slc35a1	CACACTAGCAAGGAAGATAAC
FM1_Gne	AATCCATTGACCTTCAAGAG
RM1_Gne	GATTTCACCCTTCATGCTAAC
FM1_Nans	TTTCCCTACCTGGAAAAGAC
RM1_Nans	TTCTGGTATTCCGAGATGAC
FM1_Acta1	GACATCAAAGAGAAGCTGTG
RM1_Acta1	ACTCCATACCGATAAAGGAAG

Supplementary table 1: Supplementary Table 1 shows sequence for forward and reverse primers used for the RT-PCR experiments shown in the study.

code	sex	age range	diagnosis
AFRA07	F	70-80	RA
AFRA16	F	30-40	RA
AFRA20	M	70-80	RA
AFRA23	F	50-60	RA
AFRA25	F	50-60	RA
AFRA26	M	70-80	RA
8378/17	M	50-60	RA
0542/18	M	60-70	RA
0418/18	F	60-70	RA
0563/18	F	20-30	RA
0400/18	F	60-70	RA
0485/18	M	70-80	RA
8348/17	M	70-80	OA
0466/18	M	50-60	OA
0471/18	M	50-60	OA
0438/18	M	70-80	OA
0437/18	M	40-50	OA
0538 /18	M	30-40	OA
SA012	M	50-60	RA
SA005	F	70-80	RA
SA007	M	40-50	RA
33537/19	M	50-60	RA
SA039	F	50-60	RA
SA040	M	40-50	RA
SA079	F	80-90	RA
SA132	F	50-60	RA
SA137	F	30-40	RA

Supplementary table 2: Supplementary Table 2 shows age range, gender and diagnosis for each individual patients in the study.
