# Supplementary material for: Loss of α2-6 sialylation promotes the transformation of synovial fibroblasts into a pro-inflammatory phenotype in arthritis
Source: Nat Commun. 2021 Apr 20;12:2343. doi: 10.1038/s41467-021-22365-z (PMC8058094; doi:10.1038/s41467-021-22365-z)
Supplement: Supplementary file 3 — Reporting Summary [file 41467_2021_22365_MOESM3_ESM.pdf]

nature research

Corresponding author(s): Miguel A. Pineda

Last updated by author(s): 22-02-2021

Reporting Summary

Nature Research wishes to improve the reproducibility of the work that we publish. This form provides structure for consistency and transparency in reporting. For further information on Nature Research policies, see Authors & Referees and the Editorial Policy Checklist .

Statistics

For all statistical analyses, confirm that the following items are present in the figure legend, table legend, main text, or Methods section.

n/a	Confirmed	
☐	☒	The exact sample size (*n*) for each experimental group/condition, given as a discrete number and unit of measurement
☒	☐	A statement on whether measurements were taken from distinct samples or whether the same sample was measured repeatedly
☐	☒	The statistical test(s) used AND whether they are one- or two-sided<br>*Only common tests should be described solely by name; describe more complex techniques in the Methods section.*
☒	☐	A description of all covariates tested
☐	☒	A description of any assumptions or corrections, such as tests of normality and adjustment for multiple comparisons
☐	☒	A full description of the statistical parameters including central tendency (e.g. means) or other basic estimates (e.g. regression coefficient) AND variation (e.g. standard deviation) or associated estimates of uncertainty (e.g. confidence intervals)
☐	☒	For null hypothesis testing, the test statistic (e.g. *F*, *t*, *r*) with confidence intervals, effect sizes, degrees of freedom and *P* value noted<br>*Give P values as exact values whenever suitable.*
☒	☐	For Bayesian analysis, information on the choice of priors and Markov chain Monte Carlo settings
☐	☒	For hierarchical and complex designs, identification of the appropriate level for tests and full reporting of outcomes
☒	☐	Estimates of effect sizes (e.g. Cohen's *d*, Pearson's *r*), indicating how they were calculated

Our web collection on statistics for biologists contains articles on many of the points above.

Software and code

Policy information about availability of computer code

Data collection

IF image acquisition was done with Zeiss LSM 880 confocal microscope.
Cells for phenotyping were acquired with Flow cytometry: BD FACSAria III high speed cell sorter with FACSDiva (v8.0.3)
RNAseq was performed with Illumina NextSeq™ 500 platform.
qPCR data were acquired with Quant Studio 7.0 Flex Real-Time PCR System reader.
Ultrasound machine used for guided biopsies was Mylab Twice (Esaote).
ELISA and ELLA data were collected with Tecan's Sunrise microplate reader using Magellan Software v 6.6

Data analysis

GraphPath prism 8 for OS X and Excel version 15.33 were used for statistical analysis of the smaller datasets. RNA-Seq transcriptomic data analysis required the following programs and software: Hisat2 version 2.1.0, featurecounts 1.4.6, DESeq2 package using the R bioconductor project DEbrowser version 1.18.2, STRING platform version 11.0 (string-db.org). Annotation and analysis of MS data was done with the open source bioinformatic tool Glycoworkbench version 1.1. FACs data were analysed with FlowJo 8.7.3. Confocal images were analysed with Zeiss LSM Image Browser version 4.0.

For manuscripts utilizing custom algorithms or software that are central to the research but not yet described in published literature, software must be made available to editors/reviewers. We strongly encourage code deposition in a community repository (e.g. GitHub). See the Nature Research guidelines for submitting code & software for further information.

Data

Policy information about availability of data

All manuscripts must include a data availability statement. This statement should provide the following information, where applicable:

- Accession codes, unique identifiers, or web links for publicly available datasets
- A list of figures that have associated raw data
- A description of any restrictions on data availability

The transcriptomic data, including raw sequencing data and processed read counts for each sample, have been deposited in NCBI's Gene Expression Omnibus and

are accessible through GEO Series accession number GSE162306. The source data underlying Figs. 1a, 2-9 and Supplementary Figs. 1,2,4-6 are provided as a Source Data file. Other primary data files are available from the corresponding authors on reasonable request.
Human data shown in supplemental figure 2 were obtained and analyzed from the publicly available dataset from https://immunogenomics.io/fibrotime. Raw data was generated by the laboratories of Dr. Soumya Raychaudhuri and Dr. Michael B. Brenner, accessible through GEO Series accession number GSE129488. KEGG database was accessed through R bioconductor project DEbrowser version 1.18.2.

Field-specific reporting

Please select the one below that is the best fit for your research. If you are not sure, read the appropriate sections before making your selection.

☒ Life sciences ☐ Behavioural & social sciences ☐ Ecological, evolutionary & environmental sciences

For a reference copy of the document with all sections, see nature.com/documents/nr-reporting-summary-flat.pdf

Life sciences study design

All studies must disclose on these points even when the disclosure is negative.

Sample size	In the absence or appropriate preliminary data for a power analysis in the experiments with human samples (supplementary figures), sample sizes were chosen based on the general practice in the field, as sample sizes n=5-7 suffice to reveal significant differences in similar clinical studies. Experiments in remission/active RA cells were determined by sample availability and statistical test was performed.<br>Key data obtained with mouse models were generated using at least 3 independent sample sets, 4 in the case of glycomics experiments due to the higher variability associated to glycan biosynthesis pathways. For ex-vivo experiments using expanded synovial fibroblasts from mouse models, cells were pooled from at least 3 independent mice in each replicate, both for naive and arthritic animals. When synovial tissue from arthritic mice was pooled, mice with similar clinical scores were selected (scores of 8-10, where the highest possible score would be 16). A pilot study showed that synovial tissue from 3 mice undergoing collagen-induced arthritis provided approximately 100x10E6 synovial fibroblasts, and the model typically shows 70-80% of incidence and 50-60% of incidence of high scores (>4). These data were used to calculate the required number of animals to conduct the experiments leading to groups of 7-8 mice for animals undergoing experimental arthritis and 5 mice for naive controls. Sample sizes that we chose throughout our manuscript were considered sufficient as long as the differences/no differences detected were statistically significant and results were consistent with those shown by independent techniques.
Data exclusions	No data were excluded from analysis.
Replication	All information regarding biological and experimental replicates indicated in the figure legends. Analysis of RNA-Seq transcriptomic and MS-glycomic data sets were subjected to unsupervised hierarchical clustering to avoid any biased interpretation. Unsupervised hierarchical clustering with Euclidean distance was performed on the synovial fibroblast MS-glycomic data using the heatmap.2 function of the gplots package in R. Statistical difference between naive and arthritic cells resulting from the unsupervised analysis was evaluated by one tail paired t-test, indicators of significance include*p < 0.05 and **p < 0.01. To confirm these results, findings were subsequently validated by alternative methods: flow cytometric and immunofluorescence studies using lectins with well-defined sugar specificity (Vectorlabs) and qPCR using predefined primers (KiCqStart® SYBR® Green Primers,Merk, and TaqMan® assays, Thermofisher) .
Randomization	Before starting experimental models, mice were assigned to individual groups using automatic randomised block design (Excel) under a single blind protocol. Groups were not mixed within cages to prevent microbiota transfer. Experiments were not randomised. Human RA samples were allocated from patients who fulfilled the 2010 EULAR/American College of Rheumatology classification criteria for RA (Annals of the rheumatic diseases 69, 1580–1588, 2010). Human OA samples were confirmed by radiographically in individual patients.
Blinding	Transcriptomic and glycomic data acquisition were performed in separated labs located in different facilities (Glasgow Polyomics facilities, Imperial College London) where coded samples were sent and investigators were blinded to group allocation. For the rest of the experiments in the manuscript blinding was not relevant since analysis was not exposed to subjective bias.

Reporting for specific materials, systems and methods

We require information from authors about some types of materials, experimental systems and methods used in many studies. Here, indicate whether each material, system or method listed is relevant to your study. If you are not sure if a list item applies to your research, read the appropriate section before selecting a response.

Materials & experimental systems

n/a	Involved in the study	
☐	☒	Antibodies
☒	☐	Eukaryotic cell lines
☒	☐	Palaeontology
☐	☒	Animals and other organisms
☐	☒	Human research participants
☒	☐	Clinical data

Methods

n/a	Involved in the study	
☒	☐	ChIP-seq
☐	☒	Flow cytometry
☒	☐	MRI-based neuroimaging

Antibodies

Antibodies used	Antibodies specific for the following markers: vimentin (unconjugated, goat whole antiserum, Sigma V4613 Lot 058M488TV), CD106 (Pacific Blue, clone 429, Biolegend 105722, Lot B213753), CD54 (PE, clone YN1/1.7.4, Biolegend 116107, Lot B259665), CD90 (Alexa Flour 488, clone 30-H12, Biolegend, 105316, Lot B130592), CD90 (PerCP, clone 30-H12, Biolegend, 105321, Lot B130592), CD11b (FITC, clone M1/70, Invitrogen, 11-0112-85, Lot 2041735), CD45 (PE, clone 30-F11, Biolegend 103106, Lot B269212), Podoplanin (Alexa Fluor 647, clone PMab-1, Biolegend 156204, Lot B268881), CD31 (PE, clone 390, Invitrogen, 12-0311-81, Lot 1989084). Secondary antibodies: Alexa-647 conjugated chicken anti goat IgG (Thermofisher, A21469, Lot 1882197),
Validation	Only well-validated commercial antibodies were used according to the manufacturer's instructions. Suppliers and antibodies were chosen based on how much validation information was available in their website. Antibodies provided predictable results in terms of cellular staining. Antibodies specific for CD106, CD54, CD90 and CD11b were validated previously (reference 26, Arthritis Rheumatol 66, 1492–1503, 2014), where gating strategies were provided. Anti-vimentin antibody was validated by positive staining of cytoskeletal filaments by immunofluorescence in mesenchimal cells. Antibody against CD45 showed the predictable immune population in mouse splenocytes and it detected elevated percentage of positive cells in joints of arthritic mice. Similarly, antibodies against podoplanin and CD31 revealed a elevated number of positive cells in the joints of arthritic mice, in agreement with the known pathophysiology of the Collagen-Induced Arthritis model. None of the secondary antibodies used showed any positive staining in the absence of primary antibody. In addition, the positivity of the signal for all antibodies described here have been tested by isotype controls on synovial fibroblasts.

Animals and other organisms

Policy information about studies involving animals; ARRIVE guidelines recommended for reporting animal research

Laboratory animals	Male DBA/1 mice were purchased at 7-8 weeks of age (Envigo; Bicester, UK) and housed and maintained in the Central Research Facility of the University of Glasgow
Wild animals	no wild animals were used in the study.
Field-collected samples	no field-collected samples were used in the study.
Ethics oversight	All experiments were approved by, and conducted in accordance with, the Animal Welfare and Ethical Review Board of the University of Glasgow, UK Home Office Regulations and Licenses P8C60C865, I675F0C46 and ID5D5F18C.

Note that full information on the approval of the study protocol must also be provided in the manuscript.

Human research participants

Policy information about studies involving human research participants

Population characteristics	Patients fulfilling the 2010 EULAR/ACR classification criteria for Rheumatoid Arthritis were enrolled in the study. For each RA enrolled, clinical and laboratory evaluations included the number of tender and swollen joints of 28 examined, Erythrocyte Sedimentation Rate (ESR), C-Reactive Protein (CRP) and Disease Activity Score (DAS28) were collected. Peripheral blood samples were tested for IgA and IgM-RF (Orgentec Diagnostika, Bouty-UK) and ACPA (Menarini Diagnostics-Italy) using commercial Enzyme-Linked Immunosorbent Assay (ELISA) and ChemiLuminescence Immunoassay (CLIA) methods. RA patients enrolled were naive to conventional-Disease Modifying Anti-Rheumatic Drugs (DMARDs) with active disease or in sustained clinical and imaging (Power Doppler negativity at joint ultrasound assessment) sustained remission achieved by combination therapy of Methotrexate + TNFa-inhibitor. Samples obtained from joint replacement surgery were collected from patients who fulfilled the American College of Rheumatology (formerly, the American Rheumatism Association) 1987 revised criteria for RA, and patients with radiographically confirmed OA. Mean for patient age was 56.51 ± 15.6 years, n= 27, 44% female and 56% male. All data are included in supplementary table 2
Recruitment	Human subjects selection for this study was based on identification of patients with Rheumatoid Arthritis or Osteoarthritis undergoing a clinically indicated synovial tissue biopsy or surgery procedure who had provided signed informed consent for bio-specimen storage and research use. Patients meeting these criteria were included regardless of age, gender, ethnicity and race. There was no bias in recruitment and this was not a clinical trial. RA patients who underwent US-guided minimally invasive synovial tissue biopsy at the Division of Rheumatology of the Fondazione Policlinico Universitario A. Gemelli IRCCS – Università Cattolica del Sacro Cuore (namely SYNGem cohort) were enrolled in the study. RA and OA patients who underwent joint replacement surgery were recruited at Sandwell and West Birmingham Hospitals NHS Trust, Birmingham, United Kingdom.
Ethics oversight	Each enrolled patient provided signed informed consent for study participation (Università Cattolica del Sacro Cuore Ethics Committee - 6334/15) or South Birmingham Local Ethics Committee (LREC 5735).

Note that full information on the approval of the study protocol must also be provided in the manuscript.

Flow Cytometry

Plots

Confirm that:

- [x] The axis labels state the marker and fluorochrome used (e.g. CD4-FITC).
- [x] The axis scales are clearly visible. Include numbers along axes only for bottom left plot of group (a 'group' is an analysis of identical markers).
- [x] All plots are contour plots with outliers or pseudocolor plots.
- [x] A numerical value for number of cells or percentage (with statistics) is provided.

Methodology

Sample preparation	Mouse joints were dissected to expose synovial tissue, removing skin and soft tissue and preserving the bones intact. Dissected samples were incubated with 1 mg/ml of type IV collagenase for 90 minutes at 37 degrees under continuous shaking. Samples were incubated with DNaseI (5 mg/ml) and EDTA (0.5 mM). Samples were then vortexed to release cells. Cells were centrifuged and washed with cold PBS before antibody staining. Cells were filtered through a Nitex nylon monofilament mesh just prior to being loaded onto the sorter.
Instrument	Data were acquired using a FACS BD LSRII flow cytometer. Cells were sorted using a BD FACSAria IIU.
Software	FlowJo, LLC analysis software (Tree Star/ BD)
Cell population abundance	Synovial fibroblasts were sorted using a purity sort mask. Sorted cells show a purity > 99%, determined by flow cytometric analysis of sorted populations. Furthermore, expression of vimentin was evaluated by immunofluorescence in sorted cells, being vimentin a marker of stromal cells that was not used for the sort. All cells were positive. RNA was extracted from sorted cells and subjected to RNA-Seq transcriptomic analysis. Generated datasets were used to interrogate for the presence of immune cells markers (CD45, CD3, CD19) at mRNA level, with negative results.
Gating strategy	Relevant cell populations were first gated on the basis of FSC/SSC analysis and singlet and live-dead cell discrimination using Zombie Violet staining (BioLegend, 423113). Cell populations were gated using isotype controls. Immune cells and endothelial cells were excluded based on the expression of CD45 and CD31, staining that was combined under a dump channel in PE. Synovial fibroblasts were identified by their expression of podoplanin. CD31-, CD45-, podoplanin+ cells were gated and sorted as synovial fibroblast population.

- [x] Tick this box to confirm that a figure exemplifying the gating strategy is provided in the Supplementary Information.
